# Survival and prognostic factors of patients with esophageal fistula in advanced esophageal squamous cell carcinoma

**DOI:** 10.1042/BSR20193379

**Published:** 2020-01-14

**Authors:** Xin Guan, Chao Liu, Tianshuo Zhou, Zhigang Ma, Chunhui Zhang, Bojun Wang, Yang Yao, Xiaona Fan, Zhiwei Li, Yanqiao Zhang

**Affiliations:** 1 Department of Gastrointestinal Medical Oncology, Harbin Medical University Cancer Hospital, Harbin, Heilongjiang Province, China; 2 Translational Medicine Research and Cooperation Center of Northern China, Heilongjiang Academy of Medical Sciences, Harbin, Heilongjiang Province, China

**Keywords:** ESCC, esophageal fistula, esophagomediastinal fistula, esophagorespiratory fistula, prognostic factor, survival

## Abstract

The aim of the present study was to investigate the survival and prognostic factors of patients who were with advanced esophageal squamous cell carcinoma (ESCC) and developed an esophageal fistula. The data from 221 patients with advanced ESCC developed esophageal fistula from January 2008 to December 2017 at the Harbin Medical University Cancer Hospital was retrospectively analyzed. Hazard ratios (HRs) and 95% confidence intervals (CIs) were estimated by the Cox proportional hazard models. The median survival time after a diagnosis of the esophageal fistula was calculated using the Kaplan–Meier method. We found that the pathogens infected by patients are common bacteria in nosocomial infection. Besides, the incidence rate of esophagomediastinal fistula was the highest (54.2%) in the lower third of the esophagus. Kaplan–Meier analysis revealed a median survival time of 11.00 months and a median post-fistula survival time of 3.63 months in patients who developed esophageal fistula in advanced esophageal cancer. In the univariate analysis, gender, therapies for ESCC before the development of fistula, type of esophageal fistula, treatment of esophageal fistula and hemoglobin (Hb) level were the factors with significant prognostic value. Gender, type of esophageal fistula and Hb level were identified as independent prognostic factors in further multivariate analysis. In summary, our study demonstrated that several factors are significantly related to patients with esophageal fistula and should be concerned about in clinical practice.

## Introduction

Esophageal squamous cell carcinoma (ESCC) is a malignant tumor causing serious health disorders with a 5-year overall survival (OS) rate ranging from 20 to 30% [1–3]. This is presumably due to the fact that ESCC is a rapidly progressive disease and lacks typical clinical symptoms at the early stage [4,5]. Patients with unresectable or metastatic ESCC can choose symptomatic treatment, chemotherapy, radiotherapy and concurrent chemoradiotherapy (CCRT) or sequential chemoradiotherapy (SCRT) [6,7]. However, esophageal fistula is one of the severe adverse events of patients with advanced ESCC and often develops due to disease progression and therapeutic intervention [8,9]. Esophagorespiratory fistulas and esophagomediastinal fistulas are the two most common types of esophageal fistula. The tumor itself grows and invades; it would lead to esophageal fistula when it breaks through the fibrous membrane and invades adjacent structures [10,11]. Furthermore, the development of fistulas is related to the sensitivity of the tumor to treatment. The tumor would cause esophageal fistula if it subsides too quickly or the infection affects the ability of normal tissue regeneration [12,13].

In patients with advanced ESCC, the incidence of the esophageal fistula was 10–15% [14,15]. There have been a few reports of esophageal fistula, but all of them have limitations [16,17]. Specifically, some important clinical questions such as gender differences in esophageal fistula, the prognosis of different types of fistula, types of bacteria that are susceptible to be infected by esophageal fistula patients have not been answered. Therefore, the present study aims to determine the factors associated with the prognosis of patients who are with advanced ESCC and develop esophageal fistula through long-term follow-up and statistical analysis, and to clarify the clinical features of esophageal fistula.

## Materials and methods

### Patients

In the present study, 221 patients with advanced esophageal cancer and developed esophageal fistula from 1 January 2008 to 31 December 2017, were retrospectively reviewed. The patients were selected according to the following criteria:(1) patients consistent with the World Health Organization (WHO) diagnostic criteria for esophageal cancer; (2) patients diagnosed with ESCC; (3) it was divided into II–IV according to the 7th edition of the American Joint Committee on Cancer; (4) patients developed esophagorespiratory fistulas or esophagomediastinal fistulas; (5) Karnofsky performance status (KPS) score ≥ 70. Patients were excluded depending on the following criteria: (1) patients previously treated by esophageal surgery; (2) patients having esophageal cancer with other malignant tumors; (3) patients who developed other types of esophageal fistulas instead of the above two types. The included patients underwent one of the following treatment options, including symptomatic treatment, chemotherapy, radiotherapy and CCRT or SCRT, after being staged according to the AJCC/UICC TNM staging system. Meanwhile, 200 patients with advanced ESCC were collected as a control group. This group of patients also followed the above inclusion and exclusion criteria but did not develop fistulas.

Each patient signed a written informed consent prior to treatment and all data were from an electronic medical record. The study was confirmed by the Ethics Committee of the Harbin Medical University Cancer Hospital.

### Data collection and follow-up

Esophageal fistula is described as the communication between the esophagus and adjacent organs; it is accompanied by clinical manifestations such as fever, cough, difficulty in swallowing and chest pain. The esophageal fistula was confirmed when the barium meal leaked from the esophagus in an X-ray examination or the fistula was found in esophagoscopy. Based on the location of the esophageal fistula described in the case report, different types of esophageal fistula (esophagorespiratory fistulas, esophagomediastinal fistulas) were collected in detail.

Data on the patient tumor and treatment characteristics were collected, including: gender, age, body mass index (BMI), smoking status, location and length of the tumor, esophageal stenosis and therapies for ESCC before the development of fistula. Clinical data after the occurrence of esophageal fistula such as the type and treatment of esophageal fistula, hematological indicators after esophageal fistula (leukocyte count, hemoglobin (Hb) level, albumin level) and the type of bacterial infection is included. Detailed information about the relevant parameters is shown in Table 1. The formula for BMI is the weight (kg) divided by the square (m) of the height. Esophageal stenosis was confirmed according to the digestive endoscopy report. Hematology indicators were collected within 7 days after the occurrence of esophageal fistula. Follow-up: all patients were evaluated during esophageal fistula treatment and 30 days after fistula. Patients were followed up every 3 months for the first year, then every 6 months thereafter.

**Table 1:** Clinicopathological features of patients

Characteristics	Esophageal fistula (*n*=221)	Non-esophageal fistula (*n*=200)
Age, ≤60/>60, years	150 (67.9%)/71 (32.1%)	53(26.5%)/147(73.5%)
Gender, male/female	217 (98.2%)/4 (1.8%)	186 (93.0%)/14 (7%)
Smoke, n/y	72 (32.6%)/149 (67.4%)	83 (41.5%)/117 (58.5%)
BMI, ≤20/>20, kg/m^2^	97 (43.9%)/124 (56.1%)	60 (30.0%)/140 (70.0%)
T stage, non-T_4_/T_4_	124 (56.1%)/97 (43.9%)	72 (36.0%)/128 (64.0%)
Lymph node metastasis, n/y	179 (81%)/42 (19%)	103 (51.5%)/97 (48.5%)
Distant metastasis, n/y	193 (87.3%)/28 (12.7%)	154 (77.0%)/46 (23.0%)
Tumor location, upper/mid/lower	70 (31.7%)/92 (41.6%)/59 (26.7%)	44 (22.0%)/113 (56.5%)/ 43 (21.5%)
Tumor length, <5/5–10/>10, cm	113 (51.1%)/83 (37.6%)/25 (11.3%)	111 (55.5%)/66 (33.0%)/23 (11.5%)
Esophageal stenosis, n/y	48 (21.7%)/173 (78.3%)	58 (29.0%)/142 (71.0%)
Therapies for ESCC before the development of fistula, N/R/C/CCRT or SCRT	50 (22.6%)/51 (23.1%)/13 (5.9%)/107 (48.4%)	59 (29.5%)/28 (14%)/44 (22%)/69 (34.5%)
Leukocyte count, ≤10000/>10000, mm^3^	127 (57.5%)/94 (42.5%)	174 (87.0%)/26 (13.0%)
Hb, ≤12/>12, g/dl	130 (58.8%)/91 (41.2%)	31 (15.5%)/169 (84.5%)
Albumin, ≤3.5/>3.5, g/dl	82 (37.1%)/139 (62.9%)	17 (8.5%)/183 (91.5%)
CEA, ≤5.0/>5.0, ng/ml	92 (41.6%)/18 (8.1%)	177 (88.5%)/23 (11.5%)
SCC, ≤1.5/>1.5, ng/ml	55 (24.9%)/55 (24.9%)	150 (75.0%)/50 (25.0%)
Type of esophageal fistula, ERF/EMF	145 (65.6%)/76 (34.4%)	
Treatment of esophageal fistula, ST/ES	53 (24%)/168 (76%)	

Abbreviations: CEA, carcinoembryonic antigen; EMF, esophagomediastinal fistula; ERF, esophagorespiratory fistula; ES, esophageal stenting; SCC, squamous cell carcinoma antigen; ST, symptomatic treatment.

### Data analysis

The death of an esophageal fistula patient was identified as the final event. The time of the final event, post-fistula survival time, is defined as the interval from the onset of the esophageal fistula to death or last follow-up. Besides, OS is defined as the time from the diagnosis of esophageal cancer to the date of death or the last date of follow-up.

Kaplan–Meier survival analysis was performed to construct survival curves for OS and post-fistula survival time and to calculate the median survival time. Factors associated with prognosis were assessed with univariate and multivariate analyses using the Cox regression model; the *P*-value, hazard ratio (HR) value and 95% confidence interval (CI) of each variable were obtained. *P*<0.05 was considered statistically significant. Statistical analysis was conducted using SPSS 18.0 [18].

## Results

### Patients’ characteristics

A total of 221 patients (217 men and 4 women) with esophageal fistula were included in the present study. The median age (range) was 60 (38–82) years. Among them, 78.3% of patients were diagnosed with esophageal stenosis. In the entire cohort study, 145 patients (65.6%) had esophagorespiratory fistulas and 76 patients (34.4%) had esophagomediastinal fistulas. There were 168 patients who underwent esophageal stenting and the remaining 53 patients who received symptomatic treatment. Besides, only 110 of all patients had been tested for tumor markers, including carcinoembryonic antigen (CEA) and squamous cell carcinoma antigen (SCC). General features of the esophageal fistula group and the control group are described in Table 1.

**Figure 1:**
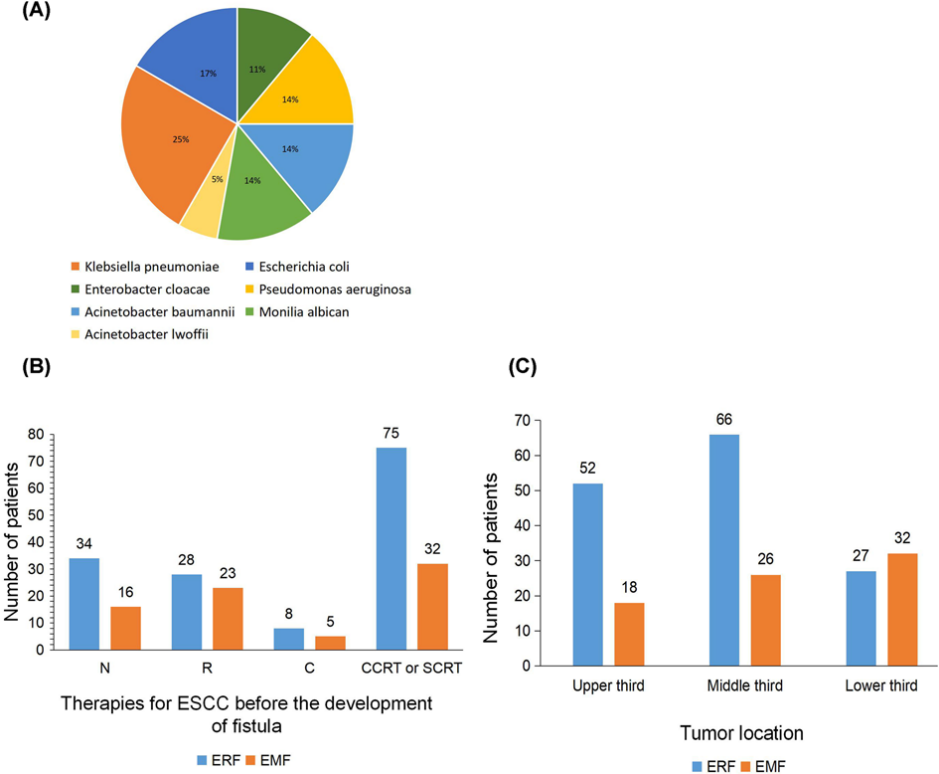
Analysis of infection distribution and clinical features of patients with esophageal fistula (**A**) Distribution of bacteria in 36 infected patients. (**B**) Number of esophageal fistulas in patients under different therapies for ESCC before the development of fistula. (**C**) Distribution of esophageal fistula in various segments of the esophagus. Abbreviations: EMF, esophagomediastinal fistula; ERF, esophagorespiratory fistula.

We found that patients who had previously received CCRT or SCRT have the largest proportion of fistula (Figure 1B). After the occurrence of esophageal fistula, 36 (16.3%) patients developed an infection, the main type of which was *Klebsiella pneumoniae* (25.0%), *Escherichia coli* (16.7%) and *Pseudomonas aeruginosa* (13.9%) (Figure 1A). Moreover, it can be shown by counting the type of esophageal fistula in each segment of the esophagus that the incidence of esophagomediastinal fistula is the highest (54.2%) of the lower third of the esophagus (Figure 1C).

### OS and post-fistula survival

**Figure 2:**
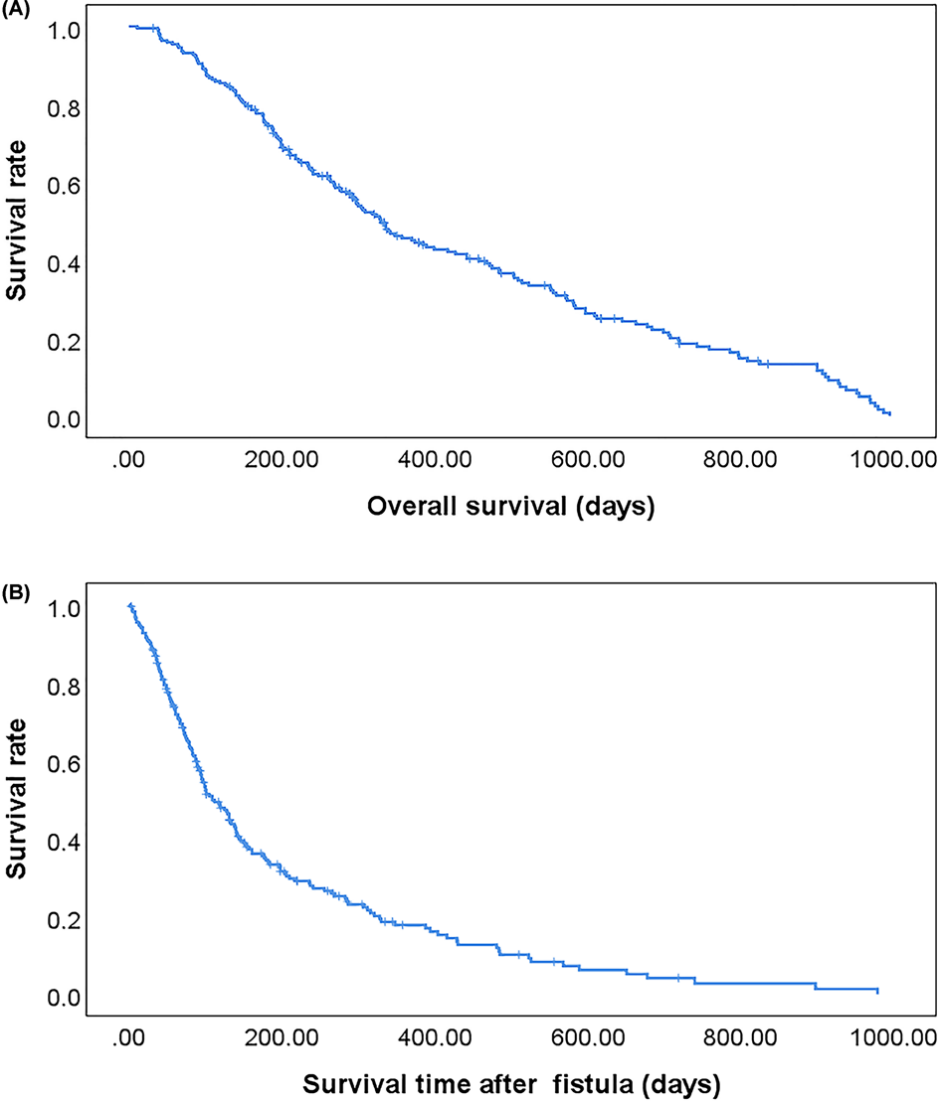
Kaplan–Meier estimates of the OS and post-fistula survival of 221 patients with esophageal fistula (**A**) OS of the 221 patients with esophageal fistulas. (**B**) Post-fistula survival of the 221 patients with esophageal fistulas.

A Kaplan–Meier survival curve and the log-rank test were used to perform survival analysis and to compare the curves of categorical variables. Overall, 178 (80.5%) died during follow-up. The median OS of esophageal cancer patients with esophageal fistula was 11.00 months; the mean OS was 14.01 months (Figure 2A). Throughout the study cohort, the median post-fistula survival time was 3.63 months (Figure 2B). Moreover, OS in the esophageal fistula group (11.00 months) was considerably shorter than that in the control group (16.30 months) (*P*<0.001; Supplementary Figure S1).

### Factors associated with survival in esophageal fistula

**Figure 3:**
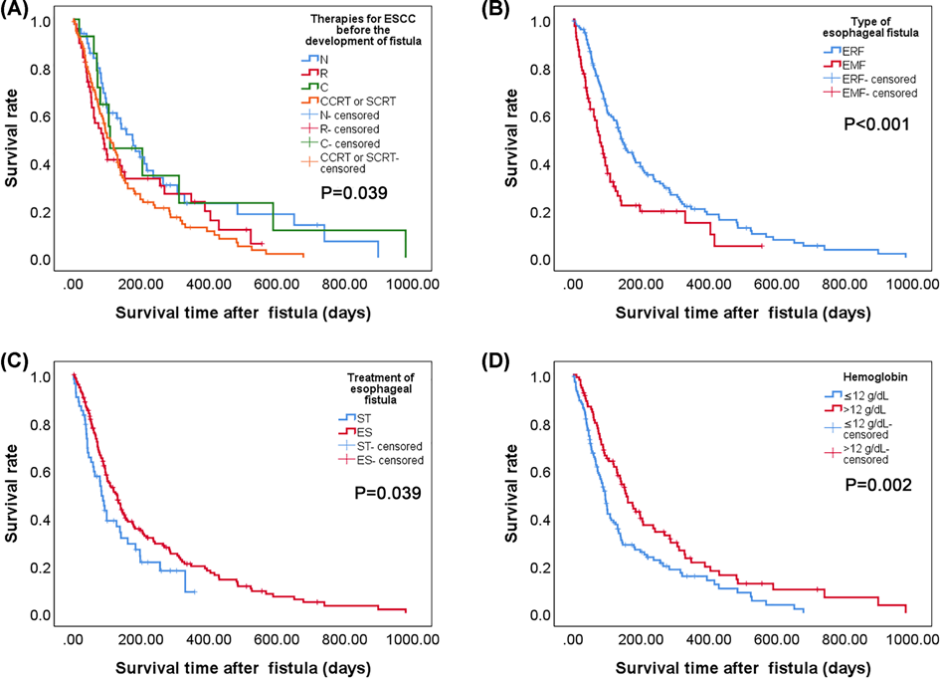
Kaplan–Meier curve for post-fistula survival (PFS) according to therapies for ESCC before the development of fistula, types of esophageal fistula, treatment of esophageal fistula and Hb level (**A**) Comparison of PFS in patients with various therapies for ESCC (*P*=0.039). (**B**) Comparison of PFS between patients with ERF and EMF (*P*<0.001). (**C**) Comparison of PFS between patients undergoing ST and ES (*P*=0.039). (**D**) Comparison of PFS between patients with Hb ≤ 12 g/dl and Hb > 12 g/dl (*P*=0.002). Abbreviations: EMF, esophagomediastinal fistula; ERF, esophagorespiratory fistula; ES, esophageal stenting; ST, symptomatic treatment.

**Figure 4:**
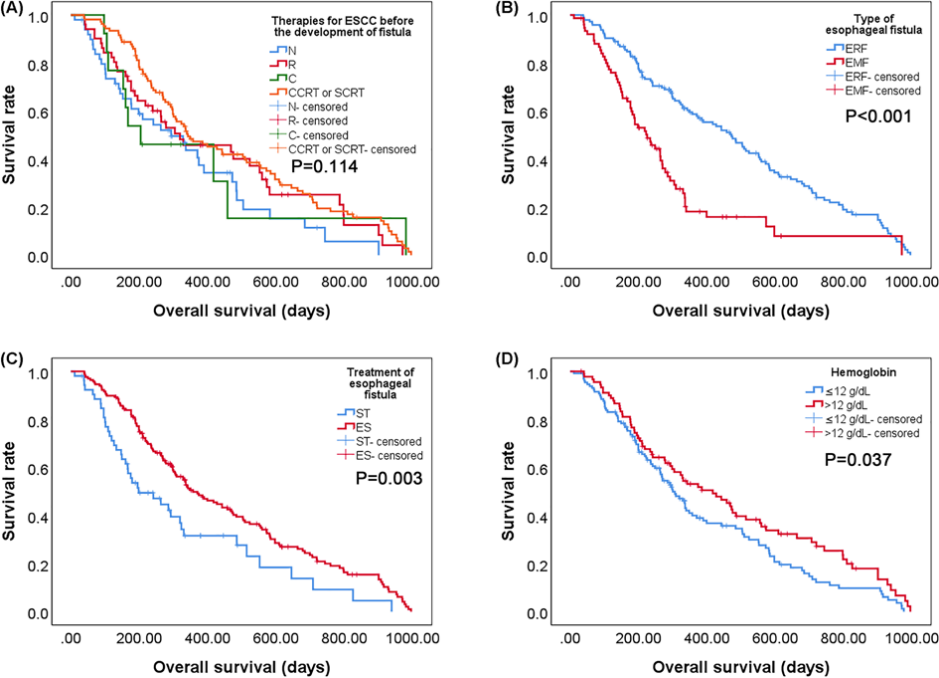
Kaplan–Meier curve for OS according to therapies for ESCC before the development of fistula, types of esophageal fistula, treatment of esophageal fistula and Hb level (**A**) Comparison of OS in patients with various therapies for ESCC (*P*=0.114). (**B**) Comparison of OS between patients with ERF and EMF (*P*<0.001). (**C**) Comparison of OS between patients undergoing ST and ES (*P*=0.003). (**D**) Comparison of OS between patients with Hb ≤ 12 g/dl and Hb > 12 g/dl (*P*=0.037). Abbreviations: EMF, esophagomediastinal fistula; ERF, esophagorespiratory fistula; ES, esophageal stenting; ST, symptomatic treatment.

It can be found by analyzing therapies for ESCC before the development of fistula in the present study that patients who had previously received radiation have a shorter survival time than patients who had not received radiation before (Figure 3A). The data demonstrated that the median post-fistula survival time of patients after the diagnosis of esophagorespiratory fistulas and esophagomediastinal fistulas was 4.80 and 2.50 months, respectively (Figure 3B). Besides, the median post-fistula survival time of patients with supportive care and esophageal stenting was 2.80 and 4.30 months, respectively (Figure 3C). In all the patients surveyed, Hb > 12 g/dl also had a better prognosis than Hb ≤ 12 g/dl (Figure 3D). The analysis of OS illustrated that the type of esophageal fistula, the treatment of esophageal fistula and the Hb level were significant factors; this result is consistent with the analysis of post-fistula survival. OS in the esophagorespiratory fistulas group was considerably shorter than in the esophagomediastinal fistula group (15.73 vs. 7.50 months; *P*<0.001) (Figure 4B). There was a statistically significant difference in OS between the 168 patients with esophageal stenting and the 53 patients with symptomatic treatment (11.90 vs. 6.60 months; *P*=0.003) (Figure 4C). And OS of patients with Hb > 12 g/dl and Hb ≤ 12 g/dl was 2.80 and 4.30 months, respectively (*P*=0.037) (Figure 4D). However, the data showed that therapies for ESCC before the development of fistula were not considered statistically significant in OS (Figure 4A).

### Prognostic factors for esophageal fistula

Univariate Cox regression analysis revealed that gender, therapies for ESCC before the development of fistula, the type of esophageal fistula, the treatment of esophageal fistula and the Hb level were prognostic factors of post-fistula survival time (*P*<0.05) (Table 2). The conclusions are consistent with those of the log-rank test. Further analysis in a multivariate Cox proportional hazards model demonstrated that gender (HR = 0.117, 95% CI: 0.016–0.857, *P*=0.035), the type of esophageal fistula (HR = 1.811, 95% CI: 1.300–2.522, *P*<0.001), and the Hb level (HR = 0.649, 95% CI: 0.477–0.884, *P*=0.006) were strongly related to the post-fistula survival time (*P*<0.05) (Table 3). It can be revealed by the univariate analysis of 110 patients who tested serum levels of tumor markers that CEA and SCC were not evaluated as significant factors (Supplementary Table S1).

**Table 2:** Univariable Cox regression analysis to identify prognostic factors of post-fistula survival

Characteristics	HR	95% CI	*P*-value
Gender, female	0.095	0.013–0.688	0.020
Age>60, years	0.827	0.597–1.145	0.252
Smoke, y	1.107	0.806–1.520	0.529
BMI>20, kg/m^2^	1.040	0.771–1.402	0.797
T stage, T_4_	0.886	0.657–1.194	0.425
Lymph node metastasis, y	1.149	0.792–1.668	0.464
Distant metastasis, y	1.342	0.877–2.054	0.176
Tumor location	0.999	0.818–1.219	0.992
Tumor length	0.964	0.775–1.198	0.738
Esophageal stenosis, y	1.400	0.961–2.039	0.079
Therapies for ESCC before the development of fistula	1.152	1.023–1.296	0.019
Leukocyte count > 10000 mm^3^	1.169	0.868–1.575	0.304
Hb > 12 g/dl	0.626	0.461–0.850	0.003
Albumin > 3.5 g/dl	0.906	0.668–1.229	0.525
Type of fistula, EMF	1.927	1.399–2.655	<0.001
Treatment of esophageal fistula, ES	0.687	0.479–0.985	0.041

Abbreviations: EMF, esophagomediastinal fistula; ES, esophageal stenting.

**Table 3:** Multivariate Cox regression analysis to identify prognostic factors of post-fistula survival

Characteristics	HR	95% CI	*P*-value
Gender, female	0.117	0.016–0.857	0.035
Therapies for ESCC before the development of fistula	1.126	0.999–1.269	0.053
Hb > 12 g/dl	0.649	0.477–0.884	0.006
Type of esophageal fistula, EMF	1.811	1.300–2.522	<0.001
Treatment of esophageal fistula, ES	0.775	0.532–1.128	0.184

Abbreviations: EMF, esophagomediastinal fistula; ES, esophageal stenting.

## Discussion

ESCC accounts for approximately 90% of 456000 incident esophageal cancers each year [19]. Esophageal fistula is one of the critical adverse events of esophageal cancer; it is closely related to the treatment of esophageal cancer and the invasion of adjacent structures such as trachea/bronchus and mediastinum [20,21]. Patients often die from nutritional failure, chest infections, mediastinal abscesses and large vessel damage, if it is not actively treated. Previous reports have focused on exploring the development of esophageal fistula and assessing risk factors for patient morbidity while reports of patients after diagnosis of esophageal fistula are rare. We found in this research of esophageal fistula patients that pathogens infected by patients are common bacteria in nosocomial infection. Previous studies have indicated that the patient’s immune function declines and is susceptible to systemic infection after developing esophageal fistula [22,23].

Male patients had a higher incidence of ESCC than female patients; the incidence ratio of male to female is close to 2.7:1 worldwide [24]. However, our study included 217 male patients and 4 female patients; besides, the number of males in esophageal fistula far exceeded that of females. In our research, females are less prone to esophageal fistula than males. In the previous studies, gender differences were noted in ESCC prognosis [24–26]. For example, Bohanes et al. [26] included 19757 patients with ESCC in a study and found that gender was shown by multivariate analysis to be independent prognostic factors. In the current study, we also found that the gender difference is an independent prognostic factor of esophageal fistula. Furthermore, the impact of estrogen on regulating metabolic and organ responses following injury has been revealed by some studies [27–29]. Therefore, females have an advantage in injury recovery, which may lead to gender differences in the occurrence and prognosis of esophageal fistula. Owing to the incidence of esophageal fistula significantly vary between males and females, the relation between gender and susceptibility to esophageal fistula in ESCC need to be further studied in future research.

Based on Kaplan–Meier analysis, our study found that patients with esophageal fistula had a median OS of 11.00 months and a median post-fistula survival time of 3.63 months. Several studies have found that the median OS of patients with esophageal fistula is approximately 8.00 months, and the mean post-fistula survival time is approximately 2.50 months [30,31]. Compared with previous studies, the survival time of esophageal fistula patients in this study is longer, which may be due to the larger number of patients in this study. In the research performed by Zhang et al. [30], 22 patients developed esophageal fistula. Besides, Kawakami et al. [31] observed in 28 patients in a retrospective analysis. Moreover, most of the of patients (27/28) received supportive care or chemotherapy in the study conducted by Kawakami et al. [31]. Conversely, most of the patients (168/221) included in this study had undergone esophageal stenting. Therefore, their survival time may be extended.

Among the parameters tested in the research, gender, therapies for ESCC before the development of fistula, the type of esophageal fistula, the treatment of esophageal fistula and the Hb level were significant factors in predicting the survival time. The implications of these factors are as follows. First, it was found in the present study that therapies for ESCC before the development of fistula was statistically significant in the univariate analysis. In a study conducted by Chen et al., the tumor shrinks during radiation therapy, resulting in the formation of a fistula in the lesion that connects the esophagus to adjacent organs [32]. Therefore, acute necrosis of cancerous tissues can be caused by radiation; therefore, the difficulty of regeneration of normal damaged tissues would be increased leading to more difficulties in healing, infection and bleeding after the occurrence of esophageal fistula. Second, the type of esophageal fistula was identified as a prognostic indicator in multivariate Cox regression analysis. Previous studies have found that esophageal fistula can cause digestive juices and bacteria to enter the mediastinum, resulting in a mediastinal abscess. The mediastinal abscess is a life-threatening emergency with a mortality rate of up to 40% due to delays in diagnosis and treatment [33,34]. However, esophagorespiratory fistula is often accompanied by an obvious cough, making it easy to be detected early. Moreover, the mediastinum itself contains a variety of important tissues and organs. Whether the formation of the esophagomediastinal fistula leads to infection or rupture of blood vessels, the consequences are extremely serious.

Third, we found that the Hb level is an independent prognostic factor of esophageal fistula. Hb level correlated with the performance status and survival of patients with various cancers [35,36]. Hb is a well-established indicator of anemia and nutritional status in ESCC [37]. Previous studies revealed the significant value of Hb predicting prognosis in patients with ESCC [37,38]. A retrospective study on ESCC showed that the OS of patients with anemia was reduced [38]. Besides, the low Hb levels may have an impact on tolerance and recovery of chemoradiotherapy [39,40]. In a series of 123 ESCC patients with neoadjuvant chemoradiotherapy, the pathologic responses of tumors could be influenced by the Hb [39]. Low Hb levels would lead to tumor hypoxia. The change can increase invasiveness and metastatic potential in ESCC [41,42]. Particularly, fistula formation often associated with tumor invasion. Therefore, whether Hb corrections can influence the prognosis of patients with esophageal fistula or not needs to be future investigated in future studies.

Finally, the application of esophageal stenting is a protective factor in the prognosis of esophageal fistula. Previous researches also demonstrated that it can cover the fistula, control the infection, restore the patency of the trachea and esophagus at the lowest cost [43,44]. Hu et al. [45] confirmed that esophageal stenting prolonged the survival time of patients with esophageal fistula and improves their life quality. Consistent with previous researches, it can be concluded that stent placement should be actively used in patients with esophageal fistula if conditions permit. The present study has some limitations that need to be acknowledged. One of the main limitations of the present study is the retrospective nature. The significance of this research is that it can contribute to quantifying the clinical characteristics, survival and prognostic factors of esophageal fistula, further improving understanding of the disease and giving follow-up treatment guides. Furthermore, the differences are difficult to be further explored and analyzed because it is difficult to accurately distinguish whether the occurrence of the esophageal fistula is caused by the treatment or disease progression. This aspect needs to be further clarified in future research.

## Conclusion

To sum up, our analysis indicated that gender, therapies for ESCC before the development of fistula, the type of esophageal fistula, the treatment of esophageal fistula and Hb level were statistically significant factors in univariate analysis. Multivariate analysis identified that male, esophagomediastinal fistula and Hb ≤ 12 g/dl were identified as poor prognostic factors. These results provide implications for communications on prognosis, treatment decisions and future investigation. Therefore, more attention should be paid to patients with esophagomediastinal fistulas and low levels of Hb. Besides, patients with esophagorespiratory/esophagomediastinal fistulas should choose esophageal stenting as a treatment method as much as possible.

## Supplementary Material

Supplementary Figure S1 and Table S1

## Abbreviations

AJCC: American Joint Committee on Cancer
BMI: body mass index
CCRT: concurrent chemoradiotherapy
CEA: carcinoembryonic antigen
CI: confidence interval
ESCC: esophageal squamous cell carcinoma
Hb: hemoglobin
HR: hazard ratio
OS: overall survival
SCC: squamous cell carcinoma antigen
SCRT: sequential chemoradiotherapy
TNM: tumor-node-metastasis
UICC: International Union Against Cancer
