# Supplementary material for: Survival and prognostic factors of patients with esophageal fistula in advanced esophageal squamous cell carcinoma
Source: Biosci Rep. 2020 Jan 14;40(1):BSR20193379. doi: 10.1042/BSR20193379 (PMC6960064; doi:10.1042/BSR20193379)
Supplement: Supplementary Figure S1 and Table S1 [file BSR-2019-3379_supp.pdf]

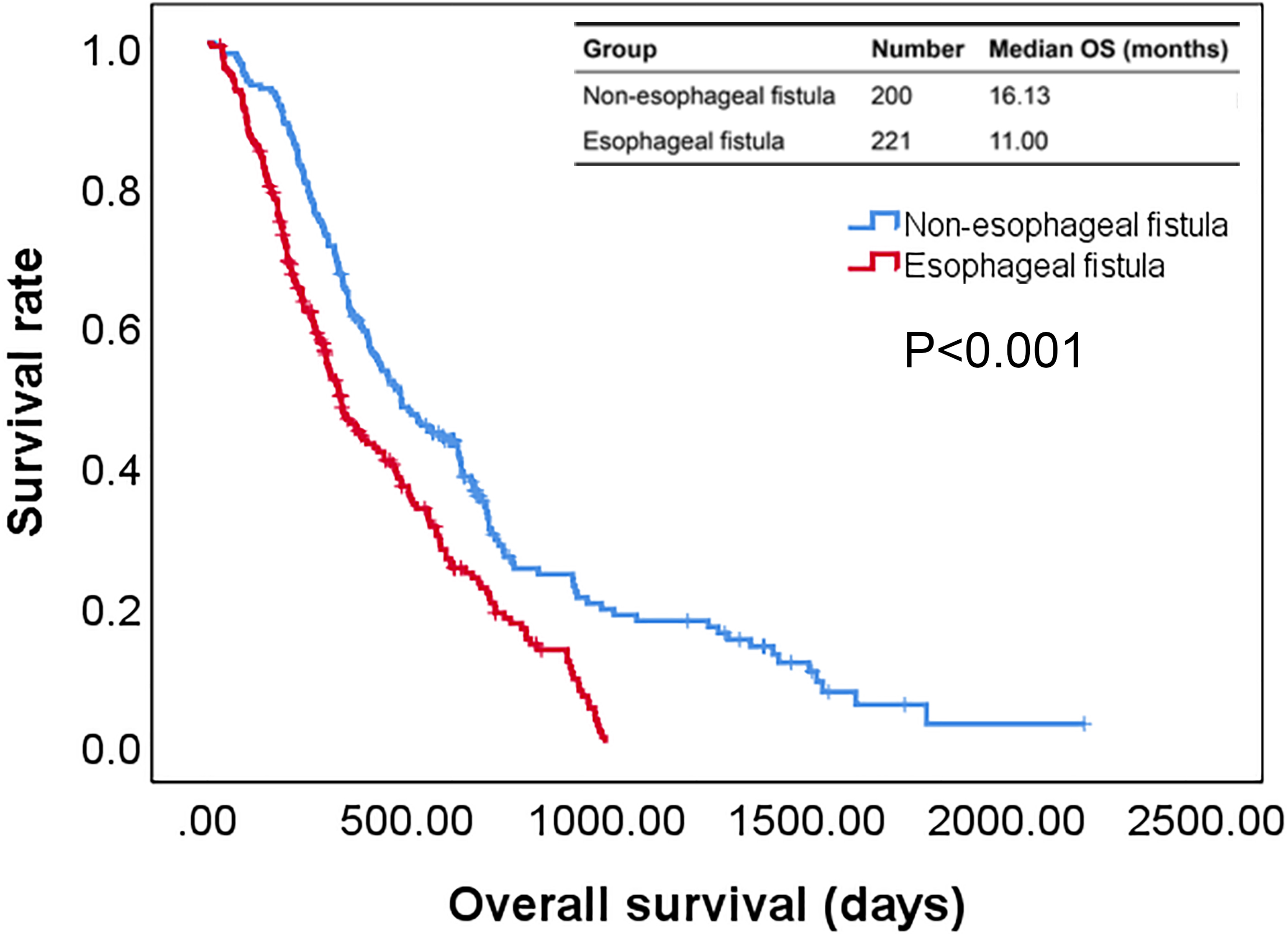

Group
Number
Median OS (months)
Non-esophageal fistula
200
16.13
Esophageal fistula
221
11.00
Non-esophageal fistula
Esophageal fistula
P<0.001
Survival rate
1.0
0.8
0.6
0.4
0.2
0.0
.00
500.00
1000.00
1500.00
2000.00
2500.00
Overall survival (days)

Supplementary Figure 1. Kaplan-Meier curve for overall survival (OS). OS in the esophageal fistula group was significantly shorter than that in the non-esophageal fistula group (p <0.001)

Supplementary Table 1. Univariate analysis was performed to evaluate the correlation between tumor markers and post-fistula survival (n=110).

HR: hazard ratio; CI: confidence interval; CEA: carcinoembryonic antigen;

SCC: squamous cell carcinoma antigen

**Characteristic**	**HR**	**95%CI**	**P-value**
CEA＞5.0 ng/ml	1.300	0.741-2.281	0.361
SCC＞1.5 ng/ml	1.104	0.726-1.679	0.642
